# Factors influencing the sustainability of digital health interventions in low-resource settings: Lessons from five countries

**DOI:** 10.7189/jogh.10.020396

**Published:** 2020-12

**Authors:** Judith McCool, Rosie Dobson, Naomi Muinga, Chris Paton, Claudia Pagliari, Smisha Agawal, Alain Labrique, Helen Tanielu, Robyn Whittaker

**Affiliations:** 1 School of Population Health, University of Auckland, New Zealand; 2 National Institute of Health Innovation, Faculty of Medical and Health Science, University of Auckland, New Zealand; 3 KEMRI-Wellcome Trust Research Programme, Nairobi, Kenya; 4 Nuffield Department of Medicine, University of Oxford, Oxford, England; 5 The Usher Institute, The University of Edinburgh, Edinburgh, Scotland; 6 Department of International Health, Johns Hopkins Bloomberg School of Public Health / JHU Global mHealth Initiative, Baltimore, Maryland, USA; 7 National University of Samoa, Apia, Samoa.; 8 Waitemata District Health Board, Auckland, New Zealand

Digital health – which includes the development and use of digital technologies and data to improve health outcomes [1] – has emerged in the past decade as a potential ‘game changer’ for enabling accessible, affordable and effective health care for all [2]. Digital health projects often involve relatively modest mobile technologies, such as short messaging service (SMS) text messaging, to address some of the most persistent barriers in health systems such as distance to services or cost [3,4]. Optimism about the potential value of digital health resulted in a flurry of pilot implementation projects undertaken over the past decade. Alas, the majority of these have failed to translate into scaled, routine services, leaving many health leaders cautious and uncertain of how to proceed [5,6]. Despite the obvious potential, there are inherent complexities that beset implementation, starting from the source of the concept (who decided on a digital intervention? on what grounds?) through to assessing the impact (who benefited? who didn’t? how do we know?).

In this paper, we draw on experiences in designing, implementing and evaluating digital health initiatives within low resource settings to identify lessons learned about factors that can influence successful and sustainable integration of digital health within local health systems. These experiences include digital health initiatives in Samoa [7], India [8], Kenya [9], Tanzania [7] and New Zealand [10] which were each undertaken in partnership with local health system providers, senior policy leads and local telecommunications providers (**Table 1**). Each intervention was ostensibly designed to ensure that digital health intervention improved the specific targeted health outcomes and could be integrated with existing health systems in a sustainable manner [5].

**Table 1:** Digital health case study characteristics

Country/populations	Name	Intervention	Origin	Evaluation	Sustainability (is it available?)
Samoa	TxtTaofitapaa	TXT Message programme for smoking cessation	NZ / Samoa Government Agreement. NZ initiated. NZ leadership, NZ funded.	Outcome evaluation (cessation measures) and process	Adapted and licenced to Ministry of Health Samoa for use. Currently unavailable
Kenya	LIFE	App programme to support training of medical and nursing staff for infant resuscitation	UK / Kenya partnership. GCRF funded.	Outcome evaluations (learning gains; clinical practice)	Distributed through Google Playstore and Apple App Store
New Zealand	TextMATCH	Text message programme to promote healthy nutrition and activity for pregnant women and their families	New Zealand	Evaluation or acceptably and adoption. Ongoing monitoring of adoption and feedback. Larger evaluation planned.	Ongoing service in 2 New Zealand health districts. Over 6000 people have signed up to date.
India	Quitnow	TXT Message for smoking cessation	WHO-ITU Be Healthy Be Mobile programme	Quit rates and quit attempts and perceptions of the programme	On-going
Tanzania	Mobile Job Aid	Mobile support tool used by community health workers to counsel women about contraception use	D-Tree, Pathfinder, USAID partnership program based on evidence based program including Counselling Strategy Plus [11]	Evaluation of acceptability, feasibility, data quality, effectiveness and costs Program has undergone several adaptations based on research findings and set up to scale in Zanzibar	Program no longer operationalised in Tanzania. Revised and delivered at scale in Zanzibar.

For the purpose of this paper, we use a general interpretation of sustainability defined as: ‘the ability to generate or gain access to the resources − financial or otherwise − needed to protect and increase the value of the content or service for those who use it” [12].

## CASE STUDY 1: SAMOA, *TXTTAOFITAPAA* (TEXT STOP SMOKE)

Experience from adapting and implementing an SMS-based smoking cessation programme in Samoa was testament to the fundamental importance of early and committed government (Ministry of Health) level involvement. Samoa, like many Pacific Islands countries, faces a growing burden of preventable non-communicable disease. With relatively low capacity in public health and prevention and high mobile network coverage and subscribership, an SMS based system to promote behaviour change was proposed and funded by the New Zealand Government. The concept was presented to the Samoa Ministry of Health within the context of a bilateral arrangement to support tobacco control in Samoa, and as part of a US and NZ agreement to support non-communicable disease (NCD) prevention in the Pacific. The adaptation process required intensive investment from the partner agencies to ensure that the final product was adapted according to smokers’ preferences [13]. We worked closely with government, civil society and the World Health Organization (WHO) to build credibility of the tool; yet ownership of the programme lay squarely with the government who held ultimate responsibility to deliver the programme. The pilot *TXTTaofitapaa* (Text Stop Smoke) intervention increased quit rates over baseline rates in a small non-randomised trial [14] a concept (using SMS support) it was appealing and the empirical evidence of impact on cessation was encouraging. Moreover, the intervention was aligned with country NCD strategy and regional priorities. However, two years on, the tool remains on the shelf ready to be implemented at scale. Human resource investment from in-country partners including local stakeholders, government, telecommunication and non-governmental organisation (NGO) sectors is more than a desirable component of any digital health initiative, it is pivotal to decisions about whether the programme should be piloted in the first place.

**Figure:**
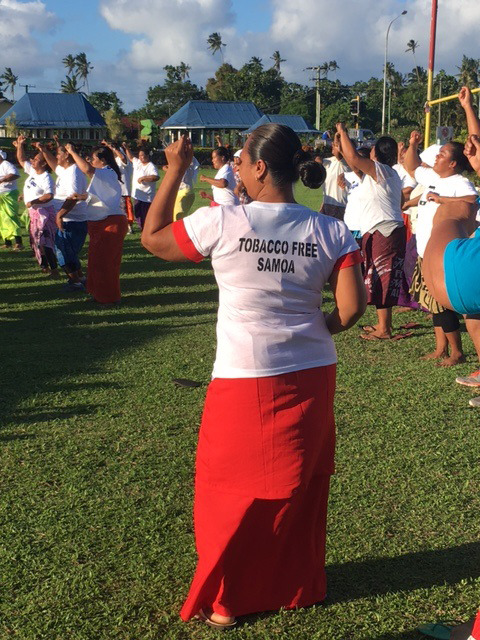
Photo: From the authors’ own collection, used with permission.

Despite efforts to build ownership from the outset of the programme, our evaluation identified a lack of locally driven investment (or ‘skin in the game’) to ensure it became embedded within the tobacco control budget and so that there was appropriate accountability for implementation. Several factors may account for the gulf between potential and actual sustainability: project fatigue (short term, externally funding projects that are not linked to clear lines of delivery and accountability), timing (a major institutional restructure was initiated at the time of potential implementation), and insufficient internal resources available to cover the hidden costs of singular initiative. Although this initiative was based on evidence of impact in other settings and appeared to have good buy-in among local stakeholders it was highly co-dependent on other government strategies or infrastructural hurdles, which can curtail implementation if the key stakeholders and strategies aren't aligned.

## CASE STUDY 2: KENYA, LIFE-SAVING INSTRUCTION FOR EMERGENCIES (LIFE)

Designing with end-users in mind is a central and widely-accepted principle of digital product development [15]. It increases the likelihood of the technologies becoming integrated into the broader system they are designed to support, for example, to complement existing teaching methods [16]. Life-saving Instruction for Emergencies (LIFE), is a 3D medical simulation smartphone and virtual reality (VR) platform developed in Nairobi, Kenya and Oxford, UK. LIFE was designed to teach health care workers in low-resource settings how to manage medical emergencies including neonatal resuscitation and the management of pneumonia in children. The team developing the LIFE app followed an approach to human-centred design (HCD) commonly employed in commercial settings called “Lean UX”. Lean UX combines approaches developed for reducing waste and increasing product quality in manufacturing, “Lean”, with contemporary human-centred design approaches to improving user experience (UX). Working closely with Kenyan nurses and doctors, the LIFE team held a series of workshops to create user personas, user story maps, and wire-frame paper-based prototypes to develop a minimal viable product (MVP). The LIFE team then iterated the MVP design in “build-measure-learn” cycles of development and testing with clinical end-users. If key features or ideas did not work for end-users, the LIFE team would “pivot” to change direction to ensure that the users’ needs were being met. An example of an important pivot was the decision to change the “look and feel” of the mobile application away from cartoon-style graphics (aimed at making the app more “fun” and game-like) to more realistic 3D graphics that users felt were better suited to the type of professional simulation training that LIFE offers. The build-measure-learn cycles revealed a range of issues that had to be addressed to ensure future sustainability of the app including: reducing the app file size; supporting a wider range of smartphones; integrating LIFE into the current teaching landscape and face-to-face training; and the importance of using an immersive VR environment to offer a safe space for clinicians to practice their skills without harming a real baby while still providing a realistic training environment [17].

The LIFE smartphone app has been downloaded by over 5400 users in Kenya and other LMICs and local partnerships with stakeholders such as the Kenya Paediatrics Association and the Nursing Council in Kenya have been established to offer Continuous Professional Development (CPD) points to health workers who successfully complete the training scenarios over a defined period. The LIFE team is also actively generating evidence by conducting to address questions that have emerged during the co-design process. In one sub-study, the LIFE team has investigated the learning experiences of clinicians using the platform and exploring how context-relevant training can be delivered [18].

Responding to the call from users for new scenarios, the LIFE project is now developing a module that will be tested in a clinical setting to support health workers in managing pneumonia in children [9].

## CASE STUDY 3: NEW ZEALAND, MATERNAL HEALTHY LIFESTYLE SUPPORT (TEXTMATCH)

Co-design and focus groups with Māori, Pacific, Asian and South Asian mothers and families were central to design of TextMATCH, a maternal and child health text message programme delivered in New Zealand. Focus group discussions with different groups determined the most appropriate programme delivery modality and allowed the programme to be tailored to the cultural characteristics and preferences of each group individually. This process of investing in the front-end of the programme – the integrity of the messages, the reflection of culturally distinct nuances in food and activity preferences underpins the importance of inclusive design and implementation planning [10,19,20]. Cultural adaptation to reality experienced by women across the different ethnic groups was an iterative and resource intensive process. Yet, this process proved essential to achieving high engagement with end users and has continually received positive feedback from users.

However, co-design may not be the single most important element of designing usable, cost-effective digital health interventions. *OL@-OR@,* a co-designed app to support Māori and Pacific communities with healthy lifestyle behaviour change found despite high engagement within the co-design process, evidence of actual and sustained behaviour changes were not detected in the cluster randomised trial of effectiveness [21]. Although disappointing, these results were a reminder that inclusive design processes alone may not be enough and the measure of success for this initiative was the process, not the outcome (product). The value lay in building partnerships that were genuinely and equally invested in the process and the considerable advantage this presents when considering digital interventions, in the future.

## CASE STUDY 4: INDIA, SMOKING CESSATION PROGRAMME (QUITNOW)

A text message-based smoking cessation programme to support smokers in India to quit was established in 2015. It was part of the World Health Organization and International Telecommunications Union (ITU) Be He@lthy Be Mobile programme which also included the support from the Ministry of Health and Family Welfare and the Ministry of Communication and Information Technology. The rationale for using a text message-based programme to promote smoking cessation was based on two key factors: an unacceptably high rate of tobacco related mortality and morbidity and an exponentially increasing rate of mobile phone use. A mCessation programme was adapted culturally and linguistically for dissemination to smokers and smokeless tobacco users in India. The initiative attracted multisector support. Early, high uptake of the programme (180 000 registered users in the first month) was reassuring. However, it became evident that method of recruitment (via phone-call) was erroneously recruiting never-smokers and people who were unsure of what they had been registered for. Despite recruitment errors, the programme was deemed feasible and effective with a 19% quit rate (no tobacco for past 30 days at 4-6 month, unvalidated). there was evidence of strong cross-sectoral support from across various government organisations. This programme has since been updated to include regional language and voice-based response system to reflect population diversity.

## CASE STUDY 5: TANZANIA, CHW SUPPORT FOR FAMILY PLANNING (FAMILY PLANNING MOBILE JOB AID)

A mobile job aid app was developed support community health workers to deliver family planning services in Dar es Salaam, Tanzania [22]. The initiative, a collaboration between Pathfinder International, D-Tree and USAID, focused on providing support to CHWs who play a key role in education, advising and facilitating access to contraception. The app was designed to serve three primary functions: a decision making tool (using an algorithm to guide CHW to counsel, screen and refer patients for family planning services); data collection and management (record routine data) and an SMS service to communicate with CHWs and their supervisors, providing updates, reminders and reports. An evaluation of the tool identified high acceptability and perceived benefits for the CHW across several areas, including increased privacy, simplification of and increased accuracy of data management [23]. Similar to most digital interventions, the app was not designed to replace human resources. Potential scale-up was feasible issues related to technology (battery charging of phones, data transmission) and cost efficiency were resolved. The programme has since been extended to CHW based in northwest Tanzania and in Zanzibar.

These case studies have demonstrated four key factors that are of importance when designing digital health interventions in low-resource contexts:

## 1. DESIGNING IN PARTNERSHIP WITH STAKEHOLDERS

Early, open and informed dialogue between all stakeholders likely to be involved with the programme implementation is a core principle for product design [15]. We suggest that three groups are particularly central: (1) Communities of end-users (groups and individuals with a vested interest in affordable, acceptable, accessible health services); (2) Government and health care stakeholders, including non-government organisation (NGOs) and civil society groups who will either be integral to the delivery of the intervention and recipients of the benefits against country/regional level priorities; and (3) Telecommunications agencies are integral to the delivery of the intervention, many considering broader economic development and commercial benefits of such partnerships with health. Accordingly, it is important to maintain an open dialog to establish and share institutional knowledge about the process of digital intervention adaptation and implementation. Adequate time needs to be devoted to the front-end of programmes, as well as throughout, to build integrated technical and human resource platforms that are resilient and adaptive. This process needs to be two-way and ongoing. All programmes described arguably sought and achieved a high level of early stakeholder involvement; the integrity of this engagement in terms of ongoing investment in the programme varied.

## 2. FOCUSING ON EQUITY THROUGH DESIGN AND EVALUATION

Digital interventions have significant potential to benefit groups who are not accessing or are not able to access traditional (face-to-face clinic-oriented) health services or health information [22]. Mobile phones are argued to be critical tools for communication for everyone, especially those with limited access to other forms of information or technology [24]. However, the digital divide is a reality for many populations, advancing ITC infrastructure might mitigate bias in access for some groups but doesn’t not overcome digital literacy. Pasifika elders living in New Zealand are suggested to be among the least able to access information via mobile, even in times of most urgent need [25]. In order to reach and adequately support low-resource, or marginalised groups, a deeper knowledge about the day-to-day functioning and values of user groups is vital. This process will identify if text messages are accessible before attempting to adapt content to prompt behaviour change. We recognise in the Pacific Islands region using social networking sites such as Facebook may have far greater reach and traction and require minimal upskilling of users [26].

Inequities in health care are not only an outcome of limited access to services, they are also perpetuated by poor quality or inappropriate services. Digital initiatives should be subject to the same measures of accountably and integrity, as with any other service, and not be a supplementary service for those unable to access services in person. Nor should digital initiatives seek to displace or override efforts to build capacity in trained health workers. Evaluation efforts measure the impact of the programme on reaching those who may benefit most as well as the sustainability [27]. A deliberate focus evidence to describe a) who gets access and b) how can this access be maintained over time are both essential measures of accountability in the evaluation process. On these grounds, initiatives which measure outcomes-based impact may miss critical evidence to support longer term programme sustainability and therefore may inadvertently exacerbate inequities. In other words, trialling a service only to identify subsequently there was no capacity for longer term investment, no detailed consideration of how it will fit into existing structures, no governance or senior leadership support, is costly, distracting and discouraging of innovation. Working as closely with the implementers as with intervention designers, is essential to enhance sustainability, and perhaps should be a prerequisite for funding in the first instance. Agawal and colleagues describe the importance of the shared funding partnership. Keeping the costs low for the investors and zero for the users, as well as monitoring unintended or hidden costs over the duration of a programme is also important.

## 3. BUILD CAPACITY, CAPABILITY AND OWNERSHIP

Irrespective of the funding source, it is essential for sustainability in the most practical terms (programmes continue to deliver and add value) that there be local ownership of these initiatives, ideally from the concept development stage. Local ownership here means that people, systems and governance structures that will be handed the responsibility to deliver initiatives after piloting are central to planning and decision making from the outset. If there is ambivalence about an initiative at the early stages, there is a high risk of the programme ever reaching scale. This reflects several core considerations. Capacity and capability building are not only important for successful implementation but even more so for ensuring that digital programmes can be maintained and updated, expanded and data extracted for improvements over the long term. For this reason, measures of ongoing capacity and capability development should be included in any evaluation of impact and sustainability. Implementation and maintenance require input (eg, official approvals) from senior managers to on-the-ground health workers (follow-up of users, content oversight) and IT support (technology glitches). Our programme evaluation in Samoa did not effectively capture the true extent of investment beyond the named staff remunerated within the budget. A recent initiative in the Cook Islands similarly relied upon Ministry of Health staff side-stepping out of usual roles periodically to support the mCessation programme. Evaluation measures should reflect the maturation of new initiatives, their interaction with difference sectors of governance and delivery systems. Digital health transitioning mechanisms (ramping up from a pilot to scaled programme) require specific consideration in the evaluation process, with clear measures and indicators of sustainability (or risk to).

Introducing a digital programme that, for example, requires users to acquire new knowledge can have unintended effects. For example, the implementation of a digital smoking cessation programme offers the immediate benefit of releasing staff from one-to-one motivational support for smokers wanting to quit and meets the requirement to deliver cessation support under WHO FCTC obligations, but it can also create a need for technical expertise to respond when, for example, pre-programmed text messages stop being sent out on schedule. Technical support is important for digital programmes to operate ‘bug-free’. Developing local capacity is important to provide this ongoing technical support. Technical troubleshooting may also require telecommunications provider input and thus shifting lines of accountability. For example, it may require task-shifting within the organisation not only to release colleagues for training but also to adapt to the role changes training can bring. Implementing a digital intervention in settings where there is no or little precedent creates a need for technical expertise, which may sway priorities and create new lines of accountability. Programme promotion, distribution and the accurate recording of baseline and follow-up measures are so essential to determine the potential value of the intervention. Once an intervention is being delivered the challenge shifts to follow-up and maintenance. Although this phase requires ongoing technical troubleshooting as needed, the importance of rigorous research and evaluation expertise within the implementation team, unless it is outsourced, is critical.

## 4. Evaluation tools need to be pragmatic

Even relatively ‘simple’ digital interventions, such as sms reminders, when implemented at scale, can impact many levels of a health system. For this reason, evaluation needs to carefully consider and prioritise impacts at the user, organisation and system level, depending on the maturity of a programme. Evidence of individual or service level impact may favour further scale-up, but without analysis of sustainability, potentially useful interventions may flounder when moving from pilot to broader implementation at a population or sub-population level.

What constitutes useful evidence of the value of a digital intervention in low-resource settings varies depending on the stakeholder. This suggests a need for nuanced impact assessments with pragmatic design and methods to capture multiple levels of (perceived and actual) value and cost. This may mean considering not only what funders determine as value for money but also what information is useful for stakeholders who carry the legacy and costs of the intervention. The Samoa mCessation programme included a comprehensive work plan with logic modelling. One stated output was a fully costed scaled up model of the programme which served the needs of both the funder and the government. A short-term output, a tailored mCessation programme for Samoa, responded to a need for additional smoking cessation support for smokers. Measures of impact may therefore prioritise behavioural outcomes over system-level or other catalytic impacts. These would have to be captured via qualitative interviewing, document analysis or temporal changes in broader determinants of health outcomes such as awareness of quitting or knowledge of access to nicotine replacement therapy, for example.

Evaluating the health system impacts of digital interventions is complex for other reasons. For example, technology is often multifaceted and can change rapidly, creating challenges for the translation of relevant evidence from one case to another. Similarly, although we argue the necessity to obtain robust evidence, interventions embedded within complex systems are not always amenable to traditional randomised controlled trials (RCTs). Mixed methods may be needed to monitor beneficial impacts and unintended negative impacts or waste. Scaled-up implementation of even highly effective interventions, such as mCessation involves several key decisions on very nuanced, practical issues. These considerations are seldom usefully informed by empirical studies of impact or reviews of the intervention, such as Cochrane review of mCessation). In the Indian mCessation project, recruitment via the ‘missed call’ system combined with mass email promotion appeared to be an easy and highly efficient method for a nationwide scaled programme. However, the evaluation identified that many people who had registered onto the programme had little understanding of what they had signed up for and had to be removed from the denominator in order to make sensible estimations of quitting smoking rates [8]. Backtracking through the protocols for recruitment was necessary to determine the reason for the high rate of ineligible participants and account for the unexpected results.

Monitoring and evaluation guides [28], reporting frameworks [29], and implementation checklists [30] can assist to ensure comprehensive consideration of the essential components or steps to successfully implement digital tools for health. In addition to these comprehensive guides, experiential knowledge gathered from working across diverse settings is a reminder about the integral role of factors such human resource capacity and capabilities, local communications (ICT) infrastructure, health literacy, cultural protocols, hierarchy and systems of practice. Using a programme logic approach, contextualised measures of digital health intervention outputs and outcomes, are necessary to calibrate expectations of realistic and meaningful outcomes. Measures of impact differ between each of the key stakeholders investing in an initiative; funders, government agencies, telcos and community groups, would inevitably measure the impact of the initiative differently. Yet, when funding is sought, the primary output (e.g., a text message programme for nutrition promotion) and outcomes (e.g., increase in knowledge of nutrition) may be only aligned with the single health issue. Capacity building across an organisation to adjust to using digital health could be a measured output given its benefit to the broader health system and a core indicator of program integrity and sustainability.

Recent investment in open source resources to support digital health interventions has been an important development and attests to the shift from design towards implementation [31]. “Global goods”, such as the DHIS2 and OpenMRS projects, support health care systems to gather and collate data from health care facilities on health care indicators to track progress towards the SDGs and support central decision making. Open source mobile platforms such as Medic Mobile and DHIS2 Tracker allow health care projects to take advantage of smartphone adoption trends in LMICs. A pragmatic approach to monitoring and evaluation of digital interventions might be the use of routine data, as collected through systems such as OpenMRS and DHIS2. Following the concept of ‘Learning Health Systems’ substituting data-driven evaluation for RCTs, which are cost-prohibitive in many LMIC contexts, holds promise [32]. The LHS concept may be particularly relevant for assessing digital interventions, which may be designed in such a way as to collect data that could be reused for evaluation and monitoring purposes (with appropriate user consent).

The success and sustainability of a digital health intervention is highly influenced by the context in which it is implemented. In low-resource settings, it is particularly important to work closely with local stakeholders from the very beginning of the design process to ensure interventions are acceptable and appropriate and that the relevant local funders and institutions will continue to support interventions if they are shown to be effective. In high-income settings, digital health may have exacerbated existing disparities in health care provisions, with those who can afford it paying for premium telemedicine consultations and remote monitoring services. However, digital health also has the potential to reduce health inequities – but only if systems are explicitly designed to address issues of equity that are prevalent in low-resource contexts. In countries with under-developed training institutions, it is necessary for digital health projects to include building local capacity in implementation planning so that local teams can take ownership following the initial development stages. In low-resource settings, where sophisticated clinical trial infrastructure would be difficult and expensive to establish, evaluations must necessarily be pragmatic and should make use of available routine data sources such as open source national health information systems and electronic medical records where possible.
